# The Path Integral Formulation of Climate Dynamics

**DOI:** 10.1371/journal.pone.0067022

**Published:** 2013-06-28

**Authors:** Antonio Navarra, Joe Tribbia, Giovanni Conti

**Affiliations:** 1 Centro Euromediterraneo sui Cambiamenti Climatici, Bologna, Italy; 2 National Center for Atmospheric Research, Boulder, Colorado, United States of America; Humboldt University, Germany

## Abstract

The chaotic nature of the atmospheric dynamics has stimulated the applications of methods and ideas derived from statistical dynamics. For instance, ensemble systems are used to make weather predictions recently extensive, which are designed to sample the phase space around the initial condition. Such an approach has been shown to improve substantially the usefulness of the forecasts since it allows forecasters to issue probabilistic forecasts. These works have modified the dominant paradigm of the interpretation of the evolution of atmospheric flows (and oceanic motions to some extent) attributing more importance to the probability distribution of the variables of interest rather than to a single representation. The ensemble experiments can be considered as crude attempts to estimate the evolution of the probability distribution of the climate variables, which turn out to be the only physical quantity relevant to practice. However, little work has been done on a direct modeling of the probability evolution itself. In this paper it is shown that it is possible to write the evolution of the probability distribution as a functional integral of the same kind introduced by Feynman in quantum mechanics, using some of the methods and results developed in statistical physics. The approach allows obtaining a formal solution to the Fokker-Planck equation corresponding to the Langevin-like equation of motion with noise. The method is very general and provides a framework generalizable to red noise, as well as to delaying differential equations, and even field equations, i.e., partial differential equations with noise, for example, general circulation models with noise. These concepts will be applied to an example taken from a simple ENSO model.

## Introduction

The equations that govern the evolution of the atmosphere and the ocean have been known for a long time and have been extensively investigated. To investigate them, several numerical methods that exploit the first order time derivatives to obtain the time evolution, have been intensely developed. The equations showed a strong sensitivity to small perturbations, both in the initial conditions as well as in the parameters defining them, giving rise to the entire field of dynamical chaos [1].

The chaotic nature of the dynamics stimulated the application of methods and ideas derived from statistics and statistical dynamics. For instance, ensemble systems are used to make weather predictions which are designed to sample the phase space around the initial condition. Such an approach has been shown to substantially improve the usefulness of the forecasts since it allows forecasters to issue probabilistic forecasts. The implicit assumption is that the presence of various sources of errors, coupled with the intrinsic sensitivity of the evolution equations to small errors [1], makes a single forecast not so useful [2], [3].

The concept has gained a large consensus because it has been shown to be relevant to various dynamical problems. Numerical experiments driven by external forcing, such as those used with prescribed SST (Sea Surface Temperature) or even prescribed concentrations of greenhouses gases in climate change experiments, have shown that the response to external forcing is still sensitive to errors, either because of uncertainties in the initial condition or in the model formulation. Ensemble experiments are now commonly used in these cases [4]–[6].

These works shifted the dominant paradigm of interpreting the evolution of atmospheric flows (and the ocean to some extent, see [7]) attributing an increasing importance to the probability distribution of the variables of interest rather than to a single representation. The ensemble experiments can be considered as crude attempts to estimate the evolution of the probability distribution of the climate variables, which is the relevant quantity for practice. Other interesting quantities, as variance and correlation functions, can be obtained from the Probability Distribution Function (PDF). The ensemble mean of temperature, for instance, cannot be considered simply as the average of the available ensemble members, but as the simplest estimation of the expectation value.

Finding an equation for the evolution of the PDF is far from trivial. Hasselmann [8] has shown that a stochastic component is consistent with the basic principles of the atmospheric/ocean dynamics and whereas other investigators [9]–[14] have shown that some aspects of the atmosphere dynamics can be described by simple models with a stochastic component. It is also possible to estimate the stochastic component from observations [15], [16].

The addition of stochastic noise to the evolution equation results in a multidimensional Langevin-like equation that can be shown to support a Fokker-Planck equation for the evolution of the probability distribution of the state vector. This result is very interesting since the Fokker-Planck equation is linear, even if the corresponding evolution equation may be non-linear. However, the Fokker-Planck equation is obtained in a phase space with the dimensions corresponding to the number of degrees of freedom of the original equations. Even a very simple general circulation model can easily have hundreds of degrees of freedom and a numerical approach is not feasible.

This paper shows that it is possible to write the evolution of the probability distribution as a functional integral of the same kind introduced by Feynman [17] in quantum mechanics, using some of the methods and results developed in statistical physics [18], [19]. The approach allows obtaining a formal solution to the Fokker-Planck equation corresponding to the Langevin-like equation of motion with noise. The method is very general and it provides a framework easily generalizable to red noise, as well as to delay differential equations, and even field equations, i.e. partial differential equations with noise. The approach has been proved useful in fields other than physics, such as polymer theory, chemistry and even financial markets [20]–[22]. There are also applications to other relevant problems in geosciences : turbulence fluids [23]–[25], Lyapunov exponents [26], data assimilation [27], or wave propagation in random media [28], [29]. The first quantum field theory formalism describing additive noise was developed by Martin, Siggia and Rose [30], by using a different kind of approach, a method similar to the canonical quantization. The path integral technique, however, is relatively less known in the field of Climatology.

In this paper, the authors attempt to solve stochastic differential equations with the Path Integral technique. This method is applied to solve a linear simple model and a non-linear one, relevant to climatological problems, to demonstrate the power of this tool. Although the technique seems involuted, it could be very easily generalized and could also be the basis for applications to field equations arising in a field theory. This method has only been used with simple linear and non-linear ENSO models, which contain only time depending variables. The aim of this paper is to stimulate interest in the path integral technique for application in the investigation of the Global Climate System. The authors' hope is to use the formalism of the field variables to face, with this technique, more complicated models, by applying this method to study general circulation models with noise.

The remainder of this paper is organized as follows. Section 0 introduces and summarizes the general theoretical foundation and Section discusses the calculation of the integrals. Section introduces the concept of Green's matrix and functions. Section introduces a discussion of perturbation expansion applied to non-linear cases. In Section, these concepts are applied to an example taken from a simple ENSO model and Section concludes.

## Methods

### The Path Integral Formulation

#### Langevin equation and probability

The systems describing the atmosphere or the ocean can be written as coupled Langevin equations: 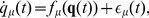
(1)where 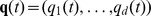 represents a trajectory in 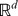 and 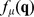 represents a differentiable function of 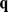. It is assumed that there are 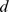 degrees of freedom, and in what follows it will be considered a Gaussian white noise 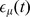. This kind of noise is characterized by its 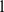-point correlation functions, the averages, that are equal to zero, and by the 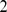-points correlation functions: 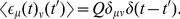
(2)


In the equation above, 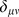 is the Kronecker delta and 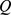 measures the strength of the correlation. For simplicity, 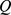 is taken as a constant, and the variances of different 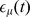 noise terms are equal. The equations above are not the most general stochastic first order differential equations. Time translation invariance has been explicitly assumed, and the same variance has been used for different variables, but those restrictions are not really limiting and it has been assumed for simplicity [31].

The Langevin equations (1) generate a time-dependent probability density function for a stochastic vector 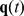, given the value of this vector at initial time, which can be written formally as: 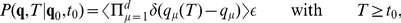
(3)in which 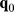 and 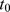 are the initial conditions, and 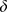 is the Dirac delta. This probability is just the ensemble average over the solutions of the Langevin equations (1); 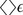 denotes an average with respect to the probability distribution of the realizations of the stochastic variables 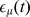. 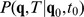 is the conditional probability to find the system in 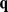 at time 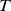 starting from the point 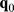 at the time 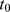. 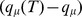 is the difference between a point of the trajectory obtained with the Langevin equation (1) at the time 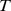, and a fixed point in the configurations space. The trajectory depends on the initial condition 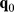, at time 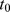. Although 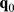 doesn't appear in the right-hand side of the equation, that expression implicitly depends on it by means 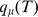.

Using the Gaussian nature of the noise, starting from the equation above, it is possible to write a Fokker-Planck equation for 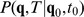, see for example [31]:
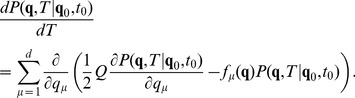
(4)


The formal solution of this equation can be written as a path integral [19]

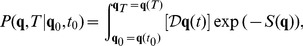
(5)where 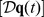 means that the integration is done over all paths 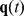 that go from 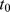 to 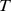. The functional 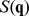 is the continuous Onsager-Machlup action which in the white noise case
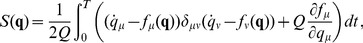
(6)for the last equation summed over repeated index it is used. The extra divergence term in the action is associated with the difficulty of defining the derivative of a stochastic process. These expressions are symbolic and, they have to be defined by a discretization rule. In fact, a functional integral is well-defined only if it is assigned a formal continuos expression and a discretization rule. The process paths, which are solutions of the Langevin equation, are continuous as 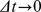, but they are not differentiable, and the ordinary rules of calculus must be modified to come up with a consistent definition. In the case of a simple additive noise, the pathologies do not show up, but if there is multiplicative noise, it is absolutely necessary to choose an interpretation. In the following, the Stratonovich interpretation will be used as the discretization rule, which allows treating the fields as differentiable, and therefore to use them in the ordinary rules of calculus. In the case of weak additive noise, the divergence term drops simplifying the action:



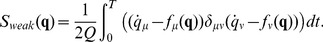
(7)Expectation values for a generic quantity 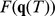 can be obtained by
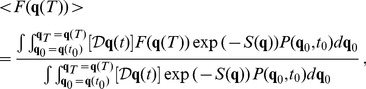
(8)where 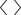 here is just a time average, and 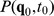 is the distribution that describes the system at the initial time 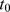. Integrating over the initial conditions using 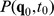, the average depends only on the point 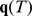. The correlation can be obtained by using a polynomial expressions of the 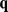 components on the functional 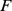.

Stochastic equations and path integrals have a mathematical meaning only if it is a discretization is associated to them. One can apply a discretization, for instance denoting the initial and final times by 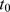 and 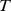, respectively, 
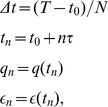
(9)with 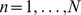. The probability distribution of the discretized noise is given by 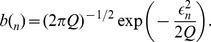



If the Langevin equation (1) is integrated in an infinitesimal time interval 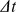, the discretized equation becomes 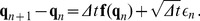
(10)


The conditional probability, that the system will be in the state 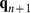 at time 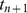 given that it was in 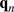 at time 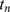, could be defined with the following symbol, 
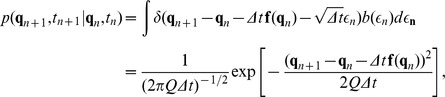
where 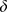 is the Dirac delta. On the right-hand side of the equation above, the only variables which appear are 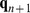, 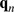, 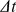, therefore it is necessary to always use a notation for which the transition probability depends explicitly on time, for instance 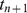 and 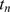. This will be more explicit, as it will soon be shown, when the summation in the action is transformed into an integral with extremes depending on the initial and final time of the transition, see Eq. (6). This means that time is a variable, not an index, and it is coherent with the fact that the PDF, which satisfies the Fokker-Planck equation, is time-dependent. In order to obtain the unconditional probability 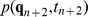, one would have to use the Kolmogorov-Chapman equation,



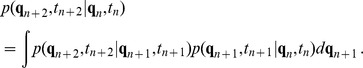
the probability for the entire path can be obtained
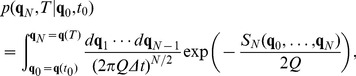
(11)where it has been defined



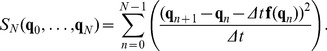
(12)The 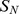 functional plays the role of the action as in classical mechanics and it is also known as the Onsager-Machlup functional. Probability cannot be exactly analytically computed for a non-linear 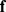, but with a linear 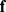 the integral is Gaussian and can be computed. From the Eq. (11) it is possible to see that 
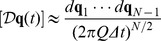
, these quantities always have to be considered in the limit approximation. There are 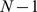 integrations over the possible intermediate values of the path, and the end points 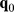, 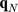 are fixed. Note that there are 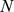 factors in the denominator of the Eq. (11), 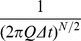, and so presumably a normalization factor will have to be introduced later, since they can be divergent when 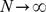. The choice of the discretization is important because the term
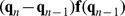
is ill-defined when the small time step limit is studied, and it must be treated carefully. It turns out that Feynman's original choice of symmetrizing the term [17] as



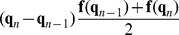
is equivalent to choosing the Stratonovich interpretation. Different continuous formal expressions exist for the functional integral, which, with the appropriate discretization rule, define the same stochastic process. The Stratonovich mean point formulation is useful to analytically treat problems. In particular it is connected to the possibility of using the usual techniques of integral calculus. With this kind of discretization, it is possible to define all the terms that in the limits become the action seen before Eq. (6). The discretization, beyond giving meaning to the expressions above, gives a recipe to explicitly compute those quantities.

#### The propagator

The probability of reaching 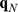 at 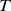 from any point 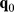 at 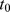, obeying the initial distribution 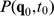, is then given by
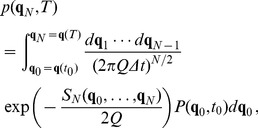
(13)which describes the evolution of the probability distribution from time 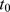 to time 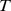. It is the solution to the Fokker-Planck equation. The final integration over 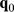 resolves the normalization issues previously mentioned and a final result is obtained. It is also possible to write Eq. (13) as
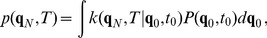
(14)where a symbol for the kernel has been introduced
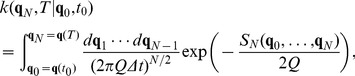
(15)that propagates the solution from time 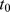 to time 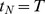; this expression is also known as the propagator. This equation is the analogous of Eq. (5) discretized.

**Figure 1:**
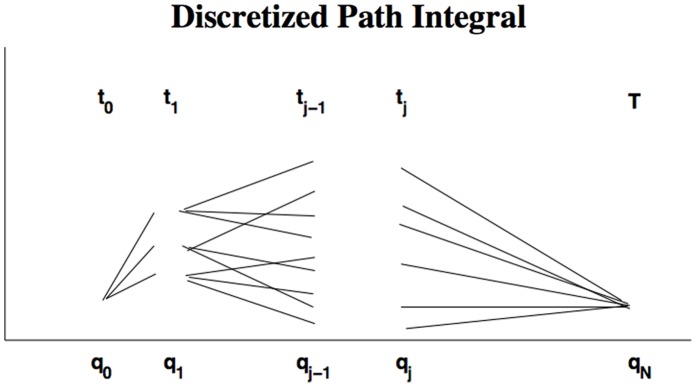
Discretization of the path integral. The initial 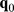 and final 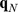 variable are not integrated over.

The concept of the path integrals recurring in these formulas is illustrated in Fig. 1. The probability of reaching 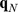 starting at 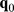 is composed by the sum of all paths that may take all possible intermediate values at intermediate times. Their contribution must be integrated for all possible values. For further details about the path integral, refer to [22].

Considering that:
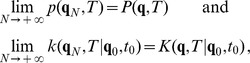
the expression for the probability in the continuous case is given by
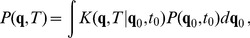
(16)and continuous time propagator from time 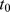 to 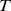 is



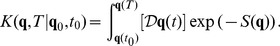
(17)Eq. (16) is the probability of finding the system in the state 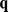 at time 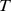, given the initial distribution 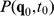 at time 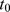.

### Calculating the Path Integral

Practically, analytically computable path integrals are rare, and they are essentially limited to Gaussian integrals, which, as previously noticed, are obtained when 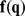 is linear. They can be analytically calculated from the discretization previously introduced only in particular cases [22]. It is possible to consider an approximate method for the computation derived from the steepest descent method (or saddle point method) [31]. The path integral is dominated by the minima of the action, which are the trajectories that minimize the action functional. It can be approximated by a series of Gaussian integral, one for each minimum of the action, considering fluctuations around these trajectories and computing the approximate integral. In this way the path integral can be separated into two simpler factors; the first one contains the stationary conditions, and the second contains a term that can be transformed, using the projection on eigenfunctions, in Gaussian integrals. If 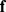 is considered as non-linear, this method results useful because it lets us use a simple perturbation expansion technique.

Let the function 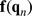 be a linear operator 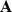. In this case the action can be written as
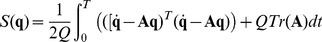
(18)and the path integrals become



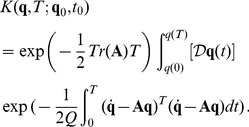
(19)Following the steepest descent method, trajectories that minimize the action must be found. However, there is a problem associated with the fact that, for a system of the present form, there are two solutions to the first order of variations, which correspond to the equation of motion without noise. The solutions correspond to the choice 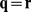, so that
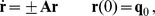
the unperturbed trajectory corresponds to the plus sign. Obviously it would be desirable to be able to investigate the perturbation around this solution, but this is complicated because the particular value of the action in this case is zero, making a traditional expansion impossible. However, as pointed out by [32], there is a method that allows the expansion along the correct solution and also satisfies both boundary conditions for the integration in the action. It is necessary to introduce a change of variables quantity 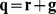, so that the action (18) can be written as
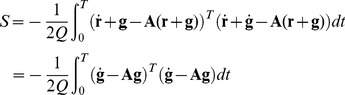
(20)because 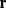 satisfies the equation of motion without noise. The boundary conditions on this expression are given by



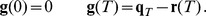



The measure of the integral does not change, since it is a linear transformation, and 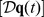 is transformed in 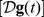 without the adjoint of a new factor in front of it. One can now substitute around an unperturbed trajectory 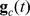 so that deviations of order 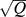 are introduced obeying the boundary conditions 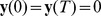


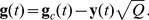
(21)


Substituting Eq. (21) in the action (20), it is
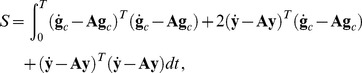
(22)and integrating by parts the various terms and using the boundary conditions, it is obtained



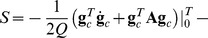


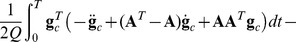
(23)

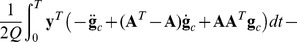


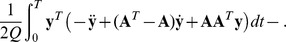



Therefore, if a 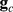 is chosen, which satisfies the equation with the given boundary conditions
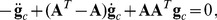
(24)the action can be divided into two parts: the explicit terms depending on the boundary conditions and implicitly on the unperturbed solution 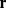, and a term that depends only on the fluctuations 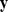,



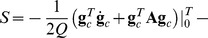


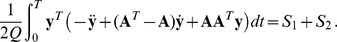



The term 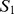 does not depend on the varying path 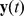 and therefore can be taken out from the integration in Eq. (19), whereas the term 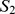 will depend only on time 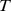, which is often called the *prefactor*. The propagator (19) can then be written as
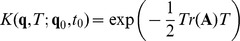


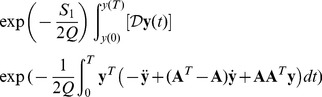
(25)with boundary conditions 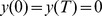. The remaining calculation can be finished by observing that the action in the paths 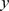 is then equivalent to a Sturm-Liouville boundary problem for the differential operator 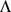




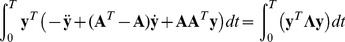
(26)The operator 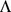 is self-adjoint and therefore has a complete orthonormal set of eigenfunctions 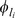 with real eigenvalues 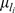, 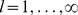, 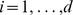. The eigenfunction and eigenvalues are d-multiple infinities as a consequence of the dimensionality 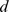 of the operator. The variables 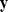 can be expanded in a series of the complete orthonormal eigenfunctions and
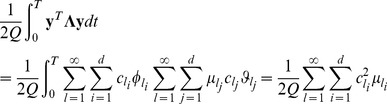
(27)


Using this approach, the path integral Eq. (17) can be written as an infinite set of Gaussian integrals over the coefficients of the expansion. A change of variables from the 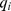
's to the 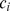
's will allow the execution of the integral. The functional path integral becomes an integral for the coefficients 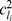, because in varying them, all possible paths are obtained. Since 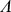 is self-adjoint, it can be diagonalized by a unitary transformation with a unit Jacobian for the change of variables, therefore the path integral measure remains the same, and the boundary conditions are satisfied by the eigenfunctions. The integral is then formed by an infinite number of Gaussian integrals, and it can be obtained that,
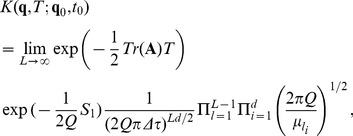
(28)or



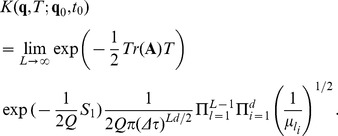
(29)The product is reduced to the inverse root of the determinant of 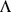. This determinant and the constant, which contain the temporal step, are usually regularized considering the ratio between this propagator and the propagator for a free evolution.

### Generating Functions

The calculation of the 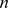-points correlation functions, that will be used to compute the correlations in the following examples, is complicated, but it can be simplified by introducing the moment generating functional
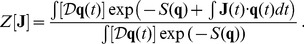
(30)


The functional derivative of the expression above
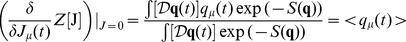
(31)provides the expectation value for the mean. Remember that the functional derivative is defined as follows



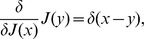
where 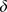 on the right-hand side is a Dirac delta, while the notation on the left is the usual notation for the functional derivation. The higher order correlations can be obtained by repeating the process:
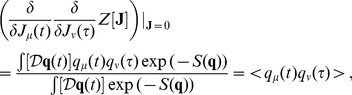
(32)and for a generic functional 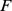, it is possible to prove that



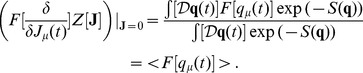
(33)The formalism of the derivation operator, appearing within the scope of 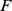, means that one has to substitute the functional derivatives in place of the usual variables on which the operator 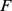 is defined as in the Eq. (32), where 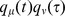 are substituted with the derivatives 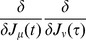.

The following paragraph shows how it is possible to compute 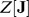 for a general case with non-linear 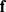 in the Langevin equations.

### Perturbation Expansions

#### Feynman diagrams

The path integral formulation adapts itself very naturally to the definition of perturbation corrections of various kinds, for example, it can be used to compute corrections to the probability distribution and to the correlation functions. In fact, because of the general complexity of the action, it will be difficult to know the exact distribution computing the integrals. Although the technique seems involuted, it can be very easily generalized and can be the basis for applications to field equations arising in a field theory.

Consider the propagator for a non-linear evolution, 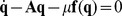, where 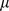 is a parameter that measures the strength of the non-linear terms,
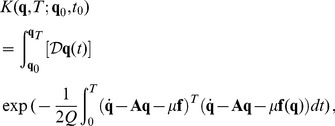
(34)that is an extension of (19). The same coordinate transformation described in Sect. (), 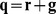, can be introduced so that the action can be written as an extension of Eq. (20)



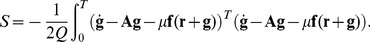
(35)Clearly the new measure 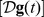 also has to be considered.

The quadratic nature of the action creates a potential problem because the expansion of the terms, according to powers of the coupling constant 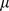, generate terms of the form 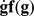 that couples state variables with derivatives. It is possible to overcome this problem using the Hubbard-Stratonovich transformation [33], [34] extended to the multidimensional case, that is a generalization of the identity
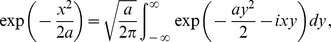
for the functional integrals. If the propagator is considered in its discretized form Eq.(15), and for each integral that appears the identity above is used when the continuos limit is restored, the propagator becomes



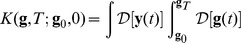


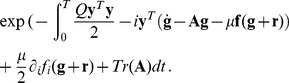
(36)


The auxiliary functions 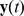 are defined over the entire time axis. This transformation introduces new integrations that can be summarized as 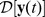. The field 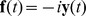 can be introduced and the trace of the linear part can be taken from the functional integrals, as it does not depend on the paths, yielding
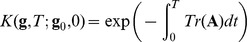


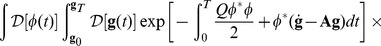


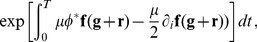
(37)or



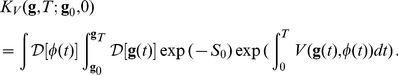
(38)The subscript 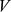 has been added to underscore the dependence of this propagator on the non-linear terms in the second exponential 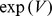, whereas the quadratic terms are contained in 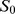. The term 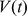 contains higher order terms in 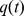 (hence in 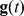 and 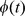) that reflect the impact of the non-linear interactions. The propagator corresponding to the quadratic part describes the evolution of the system without interaction and therefore can be described as the free evolution of the system. Usually it can be computed exactly:
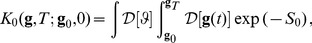
(39)whereas, in the presence of interactions, it is



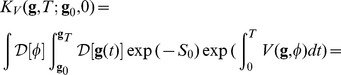


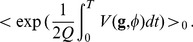
(40)


In other words, the propagator for the problem is the expected value of the interaction with respect to the probability distribution of the unperturbed, usually linear, problem. In the presence of a small coupling constant 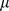, the exponential for the interaction can be expanded in series, yielding successive corrections to the free propagator
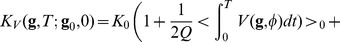


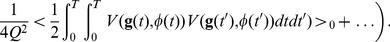
(41)


These expectation values can be computed using the generating functional Eq.(33).

#### Perturbation expansion for the correlation functions

It is useful, for the following computations, to define a scalar product as 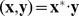, where the asterisk indicates Hermitian conjugation, 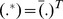. The generating function can also be written for the non-linear case using the transformed action (37). It is convenient to write it using the real vector 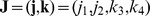 as the source term, so that
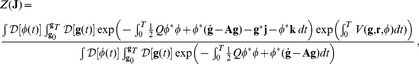
(42)where



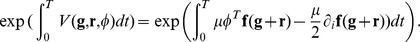



For a small coupling constant 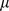, the exponential in a Taylor series can be expanded to obtain
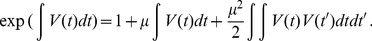
(43)


When the function of the path 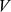 is a polynomial, every term is the expectation value of the terms of the series expansion of the exponential, and each one can be obtained by differentiating the generating function of the free evolution. The series can be formally exponentiated and written for the generating function of the non-linear case
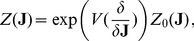
(44)analogously to Eq. (33), that must be normalized by 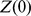. The expression for the quadratic generating function can be written as
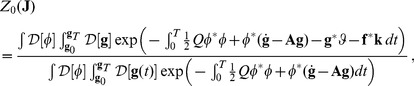
(45)where a zero subscript has been added to indicate that it is the generating function for the linear evolution. Introducing the vector 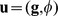, it is possible to write
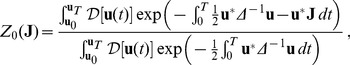
(46)where 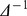 is the Hermitian operator



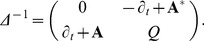
(47)It is possible to obtain an explicit form for 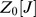 by inserting 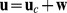, with which the numerator becomes:
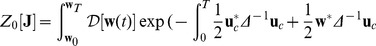


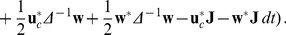
(48)


We can find 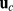 so that 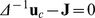, and then
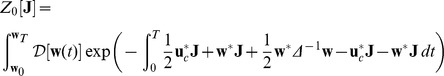


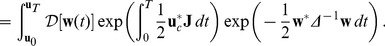
(49)


The remaining path integral over 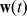 is eliminated by the normalization, therefore the generating function is given by
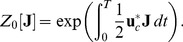
(50)


The solution 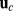 can be expressed in terms of the Green's function of the operator 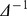,
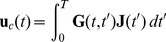
(51)and the final form of the generating function is



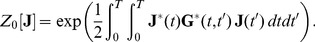
(52)This is a general expression; in fact, in the linear case a relation formally identical to the one above is obtained.

## Results and Discussion

### The Case of the ENSO

A simple model of the ENSO system based on the recharge theory was proposed years ago [35]. Following this model, ENSO can be described by a simple linear system
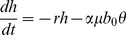


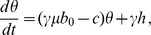
where 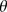 is the SST anomaly (Sea Surface Temperature) in the West Pacific and 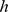 is the depth anomaly of the thermocline in the East Pacific. The parameter 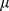 measures the strength of the interaction between the SST and the wind stress. Introducing the vector 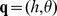, it can be written as



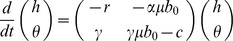



A coordinate transformation of the vector 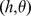 allows transforming the matrix to the standard form
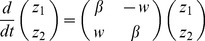
(53)


The matrix in the equation above is indicated with 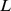.

The action for this system is given by
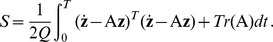



The solution without noise
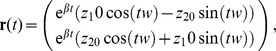
around which the action must be expanded, is represented by an exponentially modulated oscillation. The period of the oscillation is 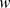 and the time scale of its exponential growth/decay is given by 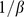. The oscillations are damped if 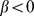, neutral oscillations occur for 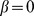 and unbounded oscillations occur in case of 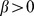. The solution of the stationarity equation (24), satisfying the boundary conditions that allow the calculation of the fluctuation prefactor, is given by the function



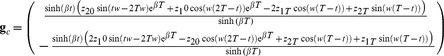
and therefore the propagator can be written, based on Eq. (25), as



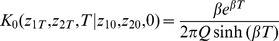


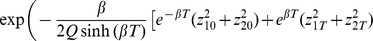


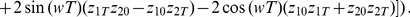
(54)


With the choice of parameters proposed in [35], 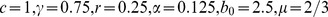, the system undergoes stable oscillations, and the entries of the corresponding matrix 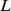 are 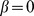 and 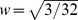. The corresponding propagator can be written as
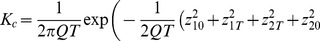


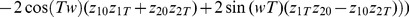
(55)


**Figure 2:**
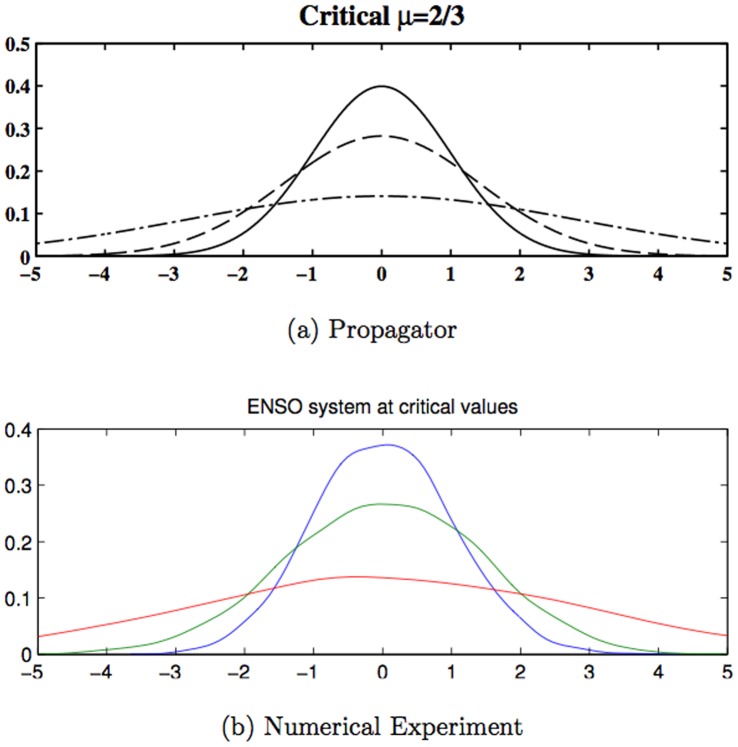
The probability distribution for the subcritical case (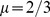) from the propagator (a) and from 2000 numerical experiment (b). The solid line corresponds to T = 1, the dashed line to T = 2 and the dot-dashed line to T = 8.

**Figure 3:**
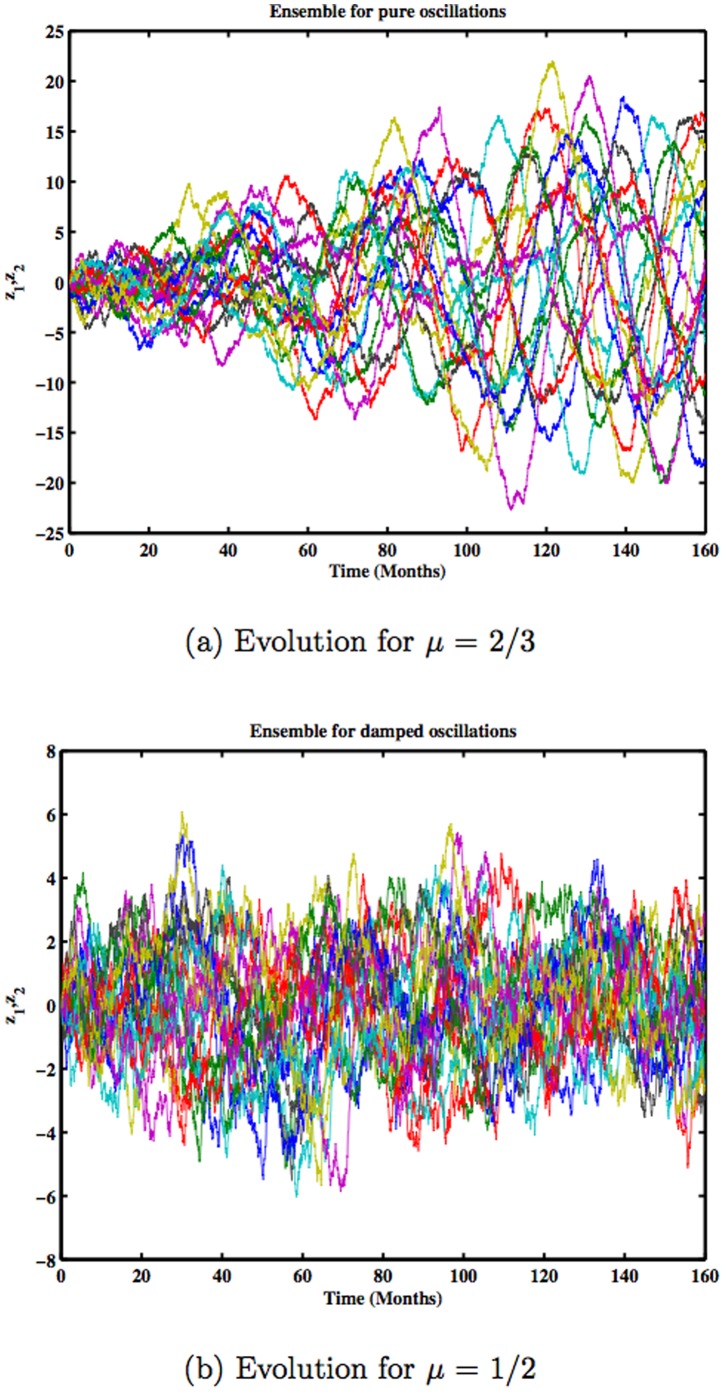
The time evolution of 10 members for the critical case
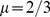
(a) and the subcritical case 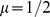 (b).

Fig. 2 shows the probability distribution, obtained for a propagator for an initial probability distribution, that is, a delta function at the origin. It is a Gaussian (the figure shows only the section for 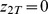), whose standard deviation increases with time. The system is analogous to a Brownian motion with the particle diffusing in the entire space. The period of the oscillation is close to 20 months and the separate members of the ensemble deviate rapidly as the system evolves. Fig. 3a shows the evolution of the individual members of the ensemble as the oscillation gains larger amplitude. The basic linear oscillation is neutral, so the stochastic fluctuations create the amplification effect, which later will result in the flattening of the probability distribution. For values of 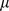 smaller than the critical value, the oscillation is damped, but the stochastic forcing can counterbalance it, permitting a statistical equilibrium. Fig. 3b shows the time evolution for the damped case, and it is possible to see how the divergence is considerably slowed down. Depending on the magnitude of the stochastic force 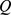, a different value of 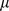 is necessary for equilibrium.

**Figure 4:**
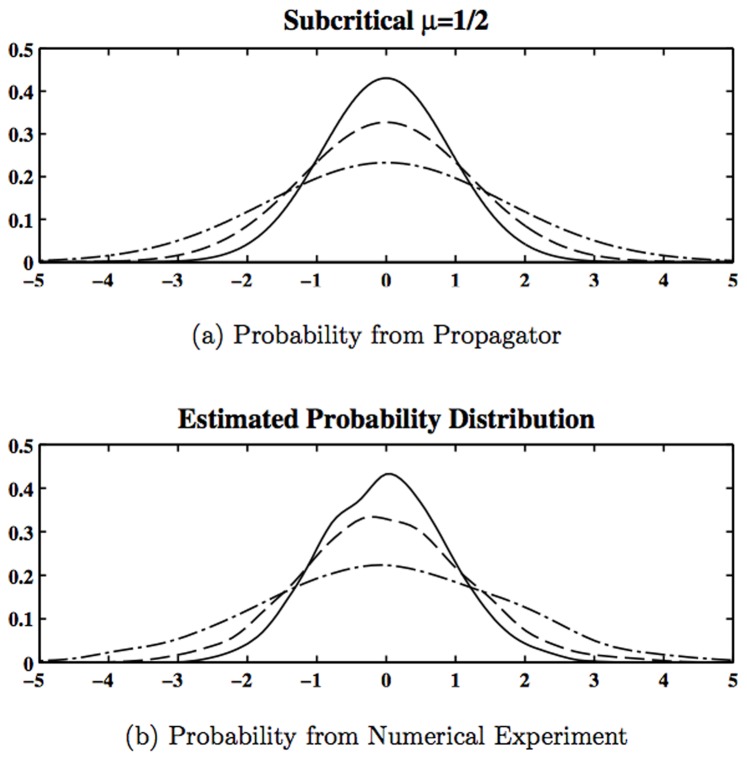
The probability distribution for the subcritical case (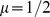) from the propagator (a) and from 2000 numerical experiment (b). The solid line correspond to T = 1, the dashed line to T = 2 and the dot-dashed line to T = 8.

The probability distribution is correctly estimated by the propagator as it can be seen in Fig. 4. The zeroth order generating function can be obtained from the Green's function as in Eq. (55). The 2-point correlation function is given by the second functional derivative of 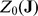,
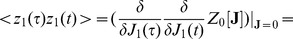


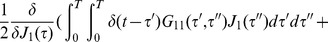


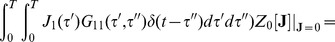


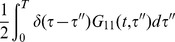


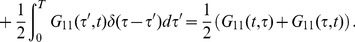
(56)


Considering more derivatives, one might also investigate higher order statistics such as the skewness. The Green's function 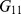 for the ENSO model in the transformed coordinates is given by
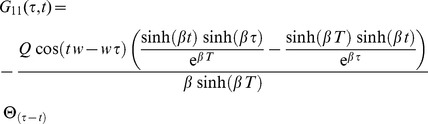


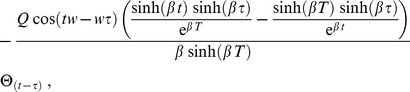
(57)where 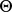 here is the sign function. In this way the standard deviation is given by equal time correlations (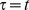)



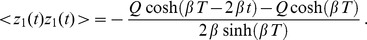



Considering the evolution for a semi-infinite domain, when 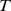 becomes very large, it will be obtained
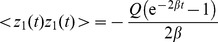
the equilibrium value



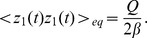



It is interesting to note that the same time correlation does not depend on the oscillating part of the solution and the frequency 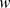 does not appear anywhere. The autocorrelation for positive lags 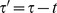 is given by
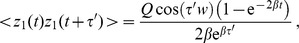
and at the equilibrium value, when 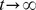,



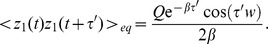



The cross-correlations in these coordinates are identically zero, but, going back to the 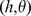 coordinates, they will recover the correlations shown in [35].

In the same paper [35], a non-linear extension of the standard model is proposed. The non-linear terms represent the negative feedback of the thermocline, and involve the strength of the coupling between the wind stress and the SST; they are cubic in 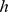 and 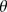. The extra term appears only in the equation for the temperature as 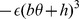



This expression can be used to get the non-linear terms in the action (36) to obtain the perturbation expansion in power of the interaction coefficient 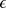, which corrects the free (linear) propagator in the presence of non-linear terms. The expansion is rather tedious and, to illustrate the point, the system will be somewhat simplified reducing the non-linear term to a simple form, obtaining a simplified version of the cubic non-linear term in the system (53) that will result in 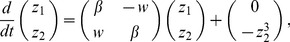
(58)where 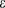, function of 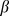, measures the strength of the non-linearity. The action for this system is given by Eq. (37), where 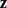 plays the role of 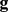. The relevant terms in the action are those deriving from 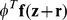 which, in this case, reduce to the interaction terms between 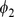 and 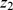, 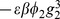. There are also terms deriving from the divergence in the action. The interaction terms are therefore given by:



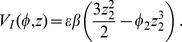



The generating function for these terms is then given by Eq. (44)
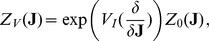
that can be expanded in power of 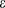,



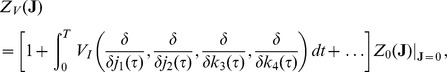
where for convenience the numbering 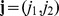 and 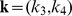 have been introduced. As one can see from Eq. (33), the functional derivatives have to be evaluated at the same time point, 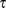, and they correspond to the powers of the dynamical variables.

As an example, the correction of the temporal covariance of 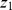 will be computed to demonstrate the approach. This covariance is given by the 2-point correlation function, as in Sect. (4),
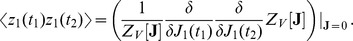



The basic rules of the functional derivation are given by
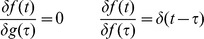
and therefore the two derivatives in 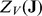 will eliminate all terms with less than two 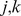, whereas the terms with a larger number of 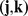 will be eliminated by the evaluation at 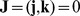. Due to these mechanisms, the derivative only selects quadratic terms in the expansion of 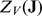. The other term in the first order expansion will be obtained by taking four derivatives, three with respect to 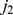, and one with respect to 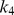. There are two terms of this kind



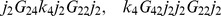



The denominator is given by the following expression
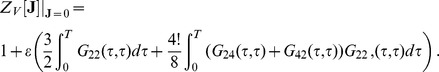



**Figure 5:**
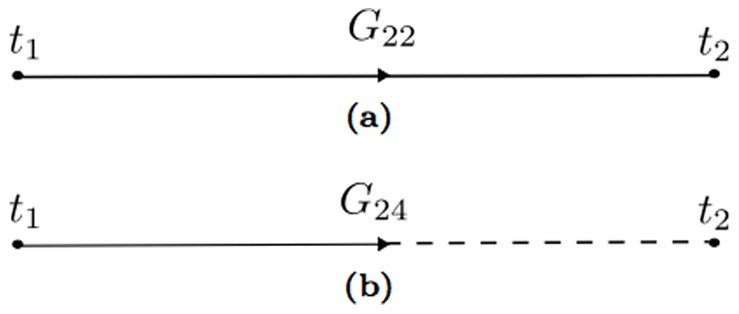
The propagators of the system: (a) the propagator for the variables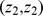 (b) the propagator for the variables 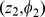. A corresponding propagator can be obtained exchanging 2 and 4.

**Figure 6:**
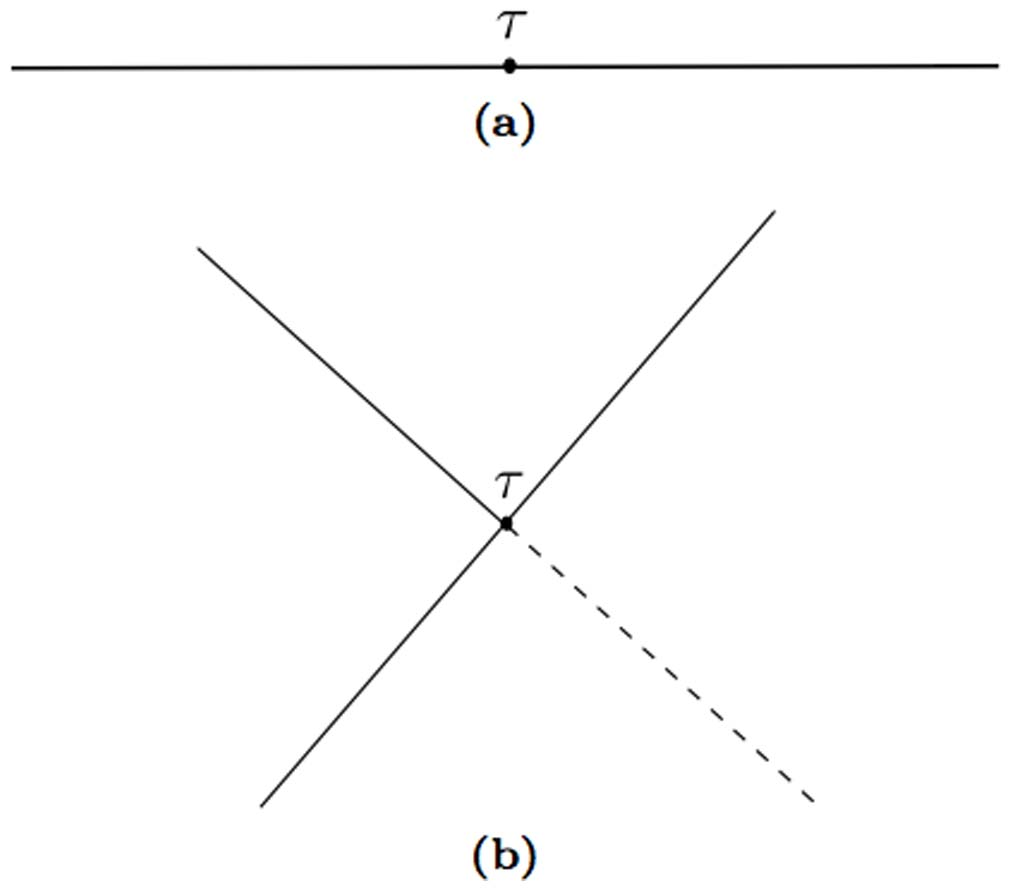
The internal vertex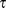. (a) for the quadratic term 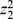, (b) for the the quartic term 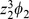.

Propagators and interactions can be graphically seen in Fig. 5 and Fig. 6. The numerator is more complicated because now there are two more derivatives. The same arguments used before now lead to the conclusion that only the terms with three Green's functions will survive. The problem is combinatorial and is well known in quantum field theory. It is essentially the same as finding all possible combinations of six points in time: the "external" points, 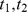, and the "internal" points 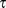 that are going to be integrated over. Depending on which of the six 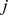 or 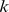 the derivatives will operate, different kinds of integrals will be generated. The zero order in 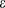 is simply 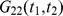, but for the first order we need to count the contribution from 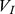. The quadratic term in 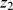 will result in
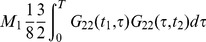
(59)

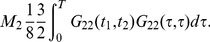
(60)


The combinatorial analysis indicates that in all there are 4 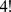 terms given by the four time points; 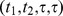 are treated, organized in such a way that 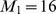 and 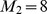. More complicated expressions are obtained from the quartic terms. In this case there are three Green's functions involved: 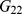, 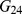 and 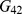. Firstly considering the combination with 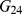, it can be seen that there are 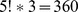 terms,
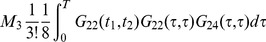
(61)

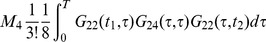
(62)

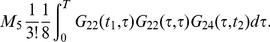
(63)with 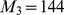, 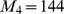, 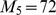. Another 360 terms will come from the symmetric terms containing 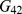. However some simplifications can be obtained because the numerator can be factored to the first order in 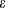 so that the normalization can be completely canceled at the denominator. The 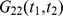 can be collected to obtain for the numerator,



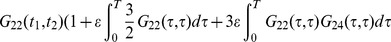


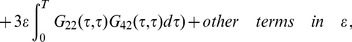
(64)or at the first order in 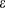




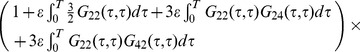


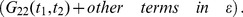
(65)


The first parenthesis cancels with the numerator and the final expression for the variance is obtained
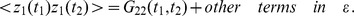



This is the unperturbed variance corrected by the non-linear terms.

The terms in the perturbation expansion can be expressed with a graphical representation via Feynman diagrams like those in Fig. 5. In this problem there are three kinds of propagators, corresponding to the matrix entries of the Green's matrix. The diagonal entries generate the propagator of the state variable 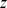, and the off diagonal terms, which turn out to be symmetric, generating the propagator connecting the state variable to the auxiliary variables 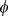. The Green's function 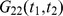 can be graphically expressed with a straight line. On the other hand, the 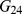 propagator can be seen as a dashed-continuos line. The points 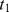 and 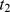 are the external lines of the graph, the time point 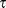 is recurring twice and is therefore special, because it has two lines that must be connected with the other point.

**Figure 7:**
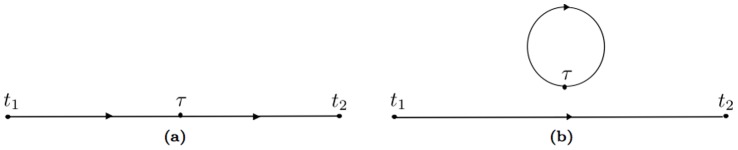
The graphical representation for the expressions (59) and (60).

The quadratic terms Eq. (60) can be graphically written as in Fig. 7. The (b) graph in the figure represents the integral where the 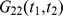 propagator can be factored out. It is an example of the fact that these kinds of terms show up graphically since they are made up of separate parts. The so-called “disconnected” graph, in this example it is the product of 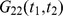 and 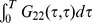.

**Figure 8:**
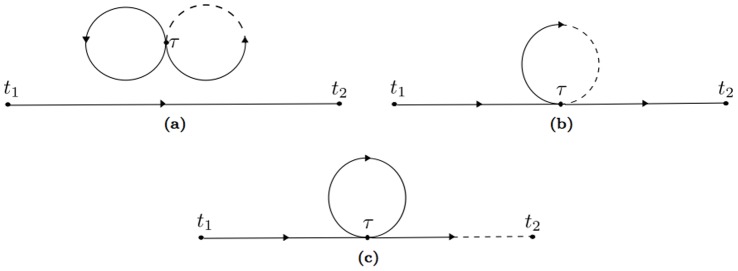
The terms of the perturbation expansion for the 2-point correlation, the variance. The full contribution can be obtained by using symmetry over all the vertices and adding the graphs obtained exchanging 2 with 4: (a) disconnected graph, corresponding to (61), (b) graph with 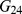 integrated over the internal vertex 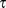, corresponding to (62), (c) graph with 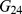 into an external point corresponding to (63).

The terms corresponding to 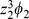 are more complicated. The internal vertex is of order four and has four lines, which must be connected with two external points. A four line vertex corresponds to the product of two Green's functions, in this case a 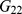 and a 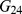, because there are only two external lines. The other two lines must be closed on themselves. The graphs are shown in Fig. 8, without showing all the possible symmetries and exchanges that produce all the 720 terms.

The disconnected graphs are the product of the component graphs, therefore the final correction to the variance or 2-point correlation can be written in the form
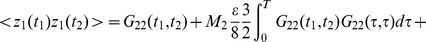


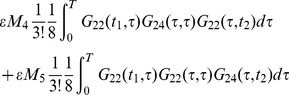
(66)


**Figure 9:**
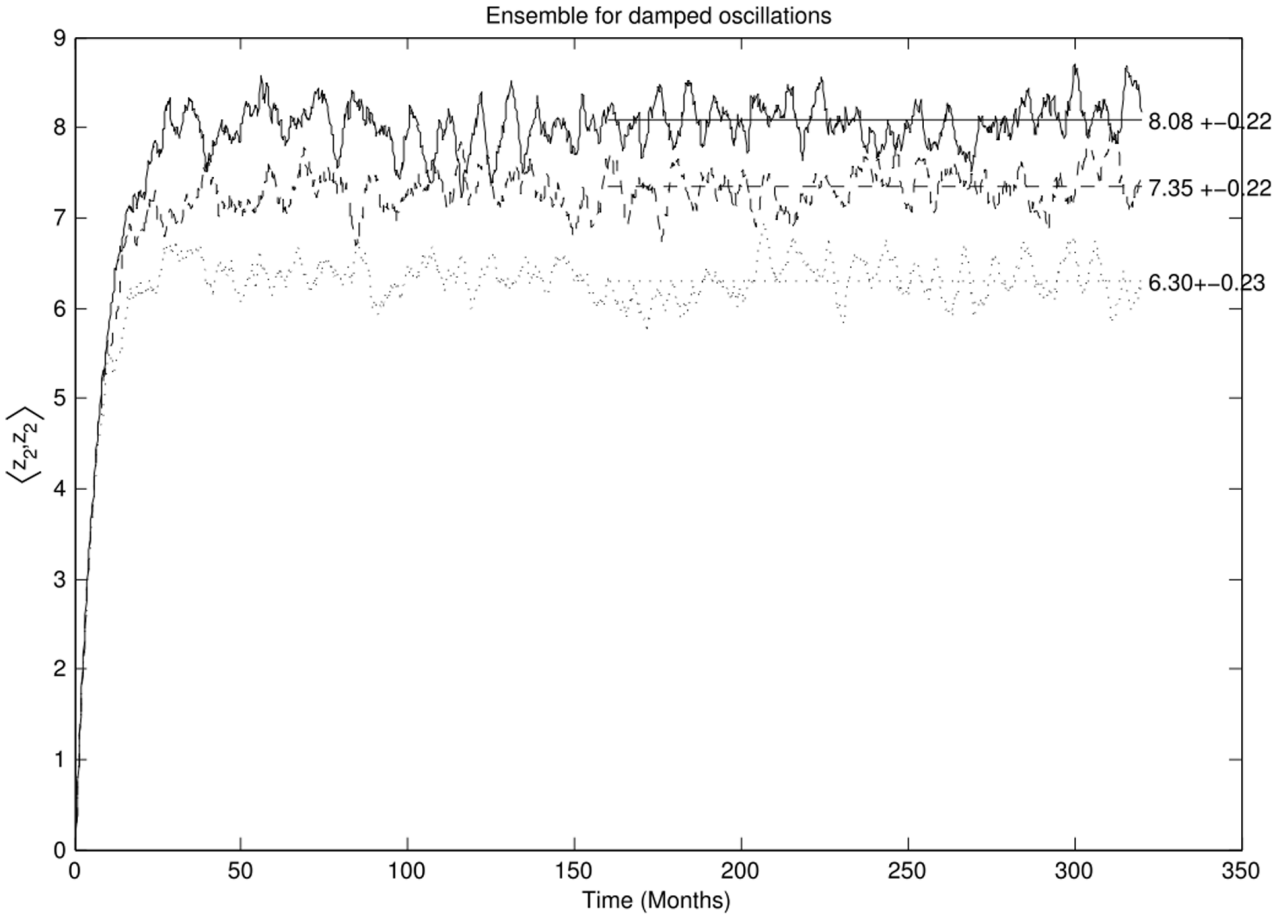
The evolution of the equal time variance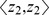 for an ensemble of 2000 simulations for the test system. The averaged variance computed after equilibration and its standard deviation is shown to the right of the figure. The solid line represents the linear system, the dashed line is the non-linear system with 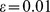 and the dotted line is the non-linear system with 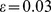.

The results are shown in Fig. 9. The figure shows the time evolution of the variance at equal times 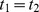 of an ensemble of 2000 numerical simulations. The solid line for the linear case concurs with the theoretical value at equilibrium, 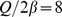, within the errors. The first order estimate of the non-linear equilibration gives 7.35 and 6.50 for 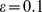 and 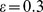 which are also in concordance with the results.

### Conclusions

This paper has shown that the path integral formulation and functional methods can be used for stochastic equations derived from the type of equation of motion that are used to describe the atmosphere and the ocean. These equations pose special complications because the evolution equations are first order in time causing an action that introduces coupling terms between the velocity terms and the forcing function.

This problem prevents a straightforward application of the method as in quantum physics, however, it can be treated by a careful consideration of the boundary conditions. Complications in higher than one dimensions can be treated using the Stratonovich-Hubbard transformation. A perturbation expansion can then be designed for non-linear cases based on the calculation of the generating function for the 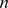-points correlation functions and Feynman diagrams can be introduced.

In this paper the path integral technique is applied to solve a linear simple model and a non-linear one, related to the Climate System, to demonstrate of the power of this tool. Although the technique seems involuted, it could be very easily generalized and could also be the basis for applications to field equations arising in a field theory. This method has only been used with linear and non-linear simple ENSO models, which contain only depending on time variables. The aim of this paper is to stimulate interest in the path integral technique to study the Global Climate System. The authors' hope is to use the formalism of the field variables to face, with this technique, more complicated models, such as applying this method to study general circulation models with noise.
